# Fracture of Bones from Trifling Violence in Cases of Cancer
*Med. Chir. Trans. Vol. XV. Part. I.


**Published:** 1829-10-01

**Authors:** 


### VIII.

### Fracture of Bones from trifling Violence in Cases of Cancer.*

Mr. Salter, a surgeon, of Poole, Dorset, has published two cases of fracture of the thigh that occurred almost spontaneously in patients affected with cancer of the breast. We shall only notice the second case, as in that, examination of the limb, post mortem, was permitted.

Mrs. Pringle, in October, 1823, had the left breast removed in Guy's Hospital, by Sir Astley Cooper, for scirrhous tumour, but in January, 1824, the disease returned in the cicatrix. In the succeeding July, whilst raising the right thigh in the attempt to get into a cart, the thigh-bone broke about three inches below the trochanter major with an audible snap, and on the 19th of October the patient died. She was 56 years of age, and had long complained of slight rheumatic pains in the affected limb, which, for about five

* Med. Chir. Trans. Vol. XV. Part. I.

months prior to the accident, had been converted into violent pain extending from the hip-joint to the knee, and appearing deep-seated, as if in the bone. The pain was worse at night, had produced great lameness, and the muscles of the thigh were extremely shrunk. The increase of pain alluded to she attributed to striking her foot against one of the stairs. A little above the patella, in front of the limb, there had been for some time a slight tumefaction, tender upon pressure, and depending, it would seem, on thickening of the periosteum. These are the important features of the case, and we proceed to the appearances found on dissection.

The muscles of the thigh were pale and shrunk—a bloody fluid escaped from the capsular ligament of the knee-joint—two or three small clots of blood were contained in the articular cavity—and, on removing the patella, an ulcer, about the size of a finger nail, was discovered in the upper and external part of its articulating surface. The head of the femur had lost its wonted smooth cartilaginous polish, but was rough and softened in its centre, whilst the thigh-bone itself was so soft that a knife could easily be pushed through it, and could readily be bent in any direction, about three inches from either extremity. It was at the upper part of this portion that the fracture had taken place, though the precise point would be difficult to determine, as there seemed to be no entire separation, as occurs in common fractures. The distortion of the limb did not arise from any overlapping, but resulted from a bending of the bone produced by the contraction of the muscles. Those in immediate contact with the trochanters and the upper half of the limb were blended together into an uniform mass, firm and semi-cartilaginous, of pale red colour, with bony spiculæ thickly dispersed through it, and puriform matter slightly tinged with blood. Corresponding to the swelling above the patella, the tendon of the cruræus was much thickened and altered in structure; pus issued from beneath it; and the periosteum was also greatly thickened and readily separated from the bone. The table of the thigh-bone at this part was almost entirely absorbed quite down to the condyles; the medullary cavity was filled with a bloody pultaceous substance; and indeed so great was the disorganization that the author abandons the task of endeavouring to describe it in words, and refers to two drawings made by his pupil, Mr. Bullar, and engraved on stone for the volume of the Transactions which contains the paper.

Mr. Salter, in the remarks he has appended to the case, seems not to be aware that the connexion between cancer and this curious condition of the osseous system has been frequently pointed out by authors on surgery. He observes that Mr. Samuel Cooper is the only author, to whose works he has access, in which this disease has been noticed. We can assure Mr. Salter, that no less a person than Mr. Cooper's celebrated namesake, Sir Astley, has particularly adverted to the fact in his published Lectures on Surgery, and mentions, if we remember right, the case of a lady whose thigh-bone broke, on merely attempting to turn in her bed. We could cite several other authors to establish the correctness of our statement, but really we believe that the occurrence of a morbid state of the bones in many cases of cancer, is too well known to require any laboured disquisition to prove it so. It was but in a very recent number of this Journal that we translated a case from the French, in which the bones of a patient who had died of cancer, fractured in every direction as the persons engaged in that office were putting her into her coffin. We are not aware, however, that many accurate dissections of the diseased bone, such as that given to his brethren by Mr. Salter, are met with in works of common circulation, and therefore we have been induced to notice it so fully here. Mr. S. deserves credit for his zeal in laying before the profession, what he conceived to be a fact not generally known.

